# A Study of Interaction Effects and Quantum Berezinskii- Kosterlitz-Thouless Transition in the Kitaev Chain

**DOI:** 10.1038/s41598-020-57796-z

**Published:** 2020-02-10

**Authors:** Sujit Sarkar

**Affiliations:** Poornaprajna Institute of Scientific Research, 4 Sadashivanagar, Bangalore, 5600 80 India

**Keywords:** Quantum mechanics, Topological insulators

## Abstract

The physics of the topological state of matter is the second revolution in quantum mechanics. We study the effect of interactions on the topological quantum phase transition and the quantum Berezinskii-Kosterlitz-Thouless (QBKT) transition in topological state of a quantum many-body condensed matter system. We predict a topological quantum phase transition from topological superconducting phase to an insulating phase for the interacting Kitaev chain. We observe interesting behaviour from the results of renormalization group study on the topological superconducting phase. We derive the renormalization group (RG) equation for QBKT through different routes with a few exact solutions along with the physical explanations, wherein we find the existence of two new important emergent phases apart from the two conventional phases of this model Hamiltonian. We also present results of a length-scale dependent study to predict asymptotic freedom like behaviour of the system. We do rigorous quantum field theoretical renormalization group calculations to solve this problem.

## Introduction

In the last decade, topological properties of quantum matter and the existence of Majorana zero modes have been the focus of intense research in quantum many-body condensed matter physics^1–16^.

Recent scanning tunneling experiments on ferromagnetic atomic chain on a superconducting substrate predict a strongly localized conductance signal around the edge, indicative of existence of zero-energy Majorana edge modes^3,17,18^. The localization is much more prominent than expected, implying a common consequence that interactions play an important role. The appearance of a zero-energy Majorana edge mode has interesting physics related to the bulk boundary correspondence of the topological state of matter. Therefore, the presence of interaction has significant effect on the topological state of matter. Thus the presence of interaction in the physical system makes it much more complex than the simple Kitaev toy model^3,19– 25^.

Apart from that, it is well known that the Coulomb interaction is always present in the solid state, either screened or weak or sometimes even stronger. Coulomb interaction leads to the different physical phenomena in quantum many-body systems, such as the Kondo effect, Mott-Hubbard transition and superconductivity to mention a few. Therefore, to get a complete picture of the topological state of a quantum many-body system, one has to consider the effect of interaction^7–9,26,27^. In the Kitaev model, the physics of interacting fermions has been not discussed or studied properly. The Kitaev model is a spinless fermion model. To consider the interaction effect, we consider the nearest-neighbour interaction.

There is another class of topological transitions in a two-dimensional system without involving any spontaneous symmetry breaking in *O*(2) or *U*(1)^7–9^ symmetry. It is well known that in a quantum phase transition, breaking of a continuous symmetry is characterized by emergence of Goldstone bosons that try to restore the full symmetry of the system^7– 9,26^. It is well known from the Mermin-Wigner theorem that the short-range interaction symmetry of many-body systems cannot be spontaneously broken at finite temperature in dimensions *d* ≤ 2^28,29^.

The emergence of Goldstone bosons is not the only mechanism for restoring the symmetry of the system. As an example, the low-temperature phase of the XY model is characterized by finite spin stiffness and by the presence of bound vortex-antivortex pairs leading to a power law decay of the spin-spin correlation function for a system with quasi-long-range order. At the Berezinskii-Kosterlitz and Thouless (BKT) transition the pairs unbind and the vortices proliferate, resulting in a state with no spin rigidity and the correlation function decays exponentially. This is the classical picture of the BKT transition^30–33^. These topological defects occur in different physical systems such as vortices in superfluid helium and dislocations in a periodic crystal. These topological defects cannot be eliminated by continuous change of the order parameter^28–30^. In quantum BKT, there is no topological transition dependent on the temperature as we observe for classical BKT. For this case the topological transition depends on the strength of the sine-Gordon coupling and the Luttinger liquid parameter.

In the present study, we explore the whole spectrum of renormalization group study of interacting Kitaev model Hamiltonian and we also study the quantum BKT (QBKT) for this model Hamiltonian explicitly for different regimes of parameter space. We show explicitly how the physics of the underlying topology shows up in different length scales of the system including the asymptotic freedom like behaviour. We present the detailed motivation below.

## Motivation and Relevance of This Study

First objective: The physics of topological states of matter is the second revolution in quantum mechanics^34^. This important concept and the resulting new important results will have significant impact not only bound to the general audience of different branches of physics but also of interest for other branches of science. This is one of the fundamental motivations to study the topological state of matter in presence of interaction.

Second objective: Both the integer quantum Hall systems and the topological insulator can be described as free fermion models. Now the question is: To what extent are such topological band structures stable with respect to the electron-electron interaction? Intrinsically, it is very difficult to theoretically describe a quantum many-body system with interactions, especially a strongly correlated one^7–9,25–27^. This motivated us to study the effect of interactions in the topological state of matter. We find that the Luttinger liquid parameter plays an important role in study of the interaction effect and it is related with the system parameters through the renormalization group equations.

Third objective: The mathematical structure and results of the renormalization group (RG) theory are a significant conceptual advancement in quantum field theory in the last several decades in both high-energy and condensed matter physics^35–38^. The need for RG is really evident in condensed matter physics. RG theory is a formalism that relates the physics at different length scales in condensed matter physics and the physics at different energy scales in high-energy physics^35–38^.

In the present study, we show explicitly how the physics underlying the topology appears at different length-scales of the system and also obtain the signature of asymptotic freedom like behaviour as also the effects of interaction. We present this length scale study not only for the complete RG equations but also for QBKT equations.

We find the exact solution for the RG flow lines and present the results along with the physical explanation.

We also ask if there is any possibility of emergent quantum phases either topological or non-topological in character.

The most successful part of the RG theory is the observation of asymptotic freedom for the high-energy physics of interacting quarks and gluons and the other is the BKT for condensed matter, i.e, the two extreme energy scale of theoretical physics. In this study we succeed in unifying these two observations of RG theory in a single framework for the topological state of matter.

## Introduction of Model Hamiltonian and the Related Basic Physics

The model Hamiltonian of the present problem is below.

1 $$\begin{array}{ccc}H & = & -t{\sum }_{i=1}^{N-1}({{c}_{i}}^{\dagger }{c}_{i+1}+h.c)+\Delta {\sum }_{i=1}^{N-1}({c}_{i}{c}_{i+1}+h.c)\\  &  & +U{\sum }_{i=1}^{N-1}(2{{c}_{i}}^{\dagger }{c}_{i}-1)(2{{c}_{i+1}}^{\dagger }{c}_{i+1}-1)-\mu {\sum }_{i}^{N}{{c}_{i}}^{\dagger }{c}_{i}\end{array}$$

${{c}_{i}}^{\dagger }({c}_{i})$ is the creation (annihilation) operator. The first term represents the hopping (*t*) between the nearest-neighbour sites, i.e., kinetic energy contribution of the spinless fermion of the model Hamiltonian. The second term (*Δ*) represents the p-wave superconducting term, the third term (*U*) represents the intersite repulsive interaction and *μ* is the chemical potential. In this model Hamiltonian, there is no on-site repulsion owing to the Pauli exclusion principle.

We first recast the model Hamiltonian in terms of Majorana fermion operators to show the deficiency to get a complete picture of topological and trivial state in presence of interactions.

2 $$H=\frac{i}{2}{\sum }_{i=1}^{N-1}[(t+\Delta ){\gamma }_{i,b}{\gamma }_{i+1,a}-(t-\Delta ){\gamma }_{i,a}{\gamma }_{i+1,b}]-U{\sum }_{i=1}^{N-1}{\gamma }_{i,a}{\gamma }_{i,b}{\gamma }_{i+1,a}{\gamma }_{i+1,b}-\frac{i\mu }{2}{\sum }_{i=1}{\gamma }_{i,a}{\gamma }_{i,b}.$$

We use the following mathematical relations during the derivation of the above model Hamiltonian in terms of Majorana fermion operators.

The analytical relations between the spinless fermion and Majorana fermion are ${c}_{i}=\frac{1}{2}({\gamma }_{i,a}+i{\gamma }_{i,b})$ and the other mathematical relations for Majorana fermion algebra are. ${{\gamma }_{i,a}}^{\dagger }={\gamma }_{i,a}$, ${{\gamma }_{i,b}}^{\dagger }={\gamma }_{i,b}$, {*γ*_*i*,*a*_, *γ*_*i*,*a*_} = 2*δ*_*i*,*j*_, {*γ*_*i*,*a*_, *γ*_*i*,*b*_} = 0, ${{\gamma }_{i,a}}^{2}={{\gamma }_{i,b}}^{2}=1$. The parametric relation between the chemical potential and the hopping integral for the transition from topological state to the non-topological state has been discussed for the Kitaev chain in the literature^5,9,12^.

At First we discuss the basic aspects of this model Hamiltonian from two perspectives. It will become clear from this analysis that there are limitations of the existing results and reveals the merit of the present study.

(A). Now we consider the first situation when *Δ* = 0 but *U* is finite.

3 $${H}_{1}=-t{\sum }_{i=1}^{N-1}({{c}_{i}}^{\dagger }{c}_{i+1}+h.c)+U{\sum }_{i=1}^{N-1}(2{{c}_{i}}^{\dagger }{c}_{i}-1)(2{{c}_{i+1}}^{\dagger }{c}_{i+1}-1)-\mu {\sum }_{i}^{N}{{c}_{i}}^{\dagger }{c}_{i}.$$

This model Hamiltonian has been studied at the mean-field level to find evidence of charge density wave (CDW) state for any finite values of *U*^37^. But the exact solution by Yang and Yang^39^ shows that the CDW phase occurs when the intersite repulsion exceeds certain values of *U*; it is of the order of unity. As we understand from the nature of the interaction, in this phase, the lowest energy state is one in which no particle has a neighbour, i.e., one of the sublattices is occupied and the other is empty. Thus every bond produces the negative energy. These two states, which break the translation symmetry of the lattice is the CDW phase.

The author of ref. ^37^ has done the RG calculation to study this problem and his result is consistent with the exact solution. We will explain explicitly the appearance of this CDW phase in our study in the presentation of the RG theory based result below.

The present problem is much more complex than that studied in the ref. ^37^ and due to the presence of the p-wave superconducting term. However we have successed in finding the exact solutions of the RG flow lines of QBKT, which we shall discuss explicitly in the QBKT section. The results we present here are based on the 2nd order and 3rd order RG method.

(B). Now we consider the second situation when *U* = 0 but *Δ* is finite. Here we express, fermionic operators in terms of Majorana fermion operators to illustrate the existing results of topological state of matter.

4 $$\begin{array}{cccc}H & = & \frac{i}{2}{\sum }_{i=1}^{N-1}[(t+\Delta ){\gamma }_{i,b}{\gamma }_{i+1,a}-(t-\Delta ){\gamma }_{i,a}{\gamma }_{i+1,b}] & -\frac{i\mu }{2}{\sum }_{i=1}{\gamma }_{i,a}{\gamma }_{i,b}.\end{array}$$

The topological state and edge mode physics have already been studied/discussed for the non-interacting Kitaev chain^3^. The parametric relation between the chemical potential and the hopping integral for the transition from the topological state to the non-topological state has already been discussed for Kitaev chain in the literature^5,9^. The basic physical aspect of the topological superconducting phase is the following. Topological superconducting phase exists with a spatial separation of two Majorana zero-energy modes, so that quantum information is stored in a highly non-local manner. But, for the conventional superconductor this interesting physics is absent.

Thus it is clear from the above discussion that the two different limits of this model Hamiltonian have been studied but the whole spectrum of the involved physics has not been covered. It is well known that it is intrinsically difficult to study a quantum many-body system with interaction. Therefore, the present problem is more challenging to solve and also to extract the emergence of different quantum phases.

## A Study of Renormalization Group: Emergence of Quantum Phases

In this section, we present the emergence of quantum phases based on RG study (Eq.  (7) and Eq.  (8)).

We do Jordan-Wigner transformation to recast the Hamiltonian (Eq.  (1)) from spinless fermion operators to the spin operators^7,9,26^.

5 $$H={\sum }_{N}[(\frac{t+\Delta }{2}){S}_{n}^{x}{S}_{n+1}^{x}+\frac{(t-\Delta )}{2}{S}_{n}^{y}{S}_{n+1}^{y}+U{S}_{n}^{z}{S}_{n+1}^{z}-\mu {S}_{n}^{z}].$$

Here *S* operators are the spin-1/2 operator. Finally we obtain the model Hamiltonian in terms of the XYZ-spin model. This model has quite a few important results in the literature^7,8,26,37^ from the perspective of magnetism but the present study and results are from the perspective of effect of the interactions on topological state of matter, This is clear from the coupling terms *U* and *Δ* which are not related to the magnetic properties of the system.

One dimensional fermionic systems are not solvable by the Fermi liquid theory due to the infrared divergence of certain vertices. An alternative theory called Tomonaga-Luttinger liquid theory has been constructed to describe the one dimensional electronic system^26^. Hence we mention very briefly the nature of the different aspects of Luttinger liquid physics to emphasize the richness of the physics of the helical Luttinger liquid. We express our model Hamiltonian in terms of two dual fields *θ*(*x*) and *ϕ*(*x*), which bosonized the Hamiltonian. The fermionic fields for right (*R*) and left (*L*) movers of a one dimensional quantum many body system are ${\psi }_{RL,\uparrow /\downarrow }(x)=\frac{1}{2\pi \alpha }{\eta }_{R,\uparrow }\,{e}^{i\sqrt{4\pi }{\varphi }_{R,\uparrow \downarrow }(x)}$, where *η*_*L*∕*R*_ is the Klein factor to preserve the anticommutivity of the fermionic field which obeys Clifford algebra^7,8,26^. These two fields are related by the relations, *ϕ*(*x*) = *ϕ*_*R*_(*x*) + *ϕ*_*L*_(*x*) and *θ*(*x*) = *θ*_*R*_(*x*) + *θ*_*L*_(*x*).

Finally, we express Hamiltonian (*H*) in terms of bosonic field operators along with the Luttinger liquid parameter (*K*)(please see the “Methods” section for detailed derivations of the bosonized Hamiltonian, specially the point *K* where enters in the model Hamiltonian).

6 $$H={H}_{0}+\frac{\Delta }{2}\int cos(2\sqrt{\frac{\pi }{K}}\theta (x))dx+U\int cos(4\sqrt{\pi K}\varphi (x))dx-\mu \sqrt{\frac{K}{\pi }}\int ({\partial }_{x}\varphi (x))dx,$$

where ${H}_{0}=\frac{v}{2}\int [{({\partial }_{x}\theta )}^{2}+{({\partial }_{x}\varphi )}^{2}]dx$ is the non-interacting part of the Hamiltonian and *v* is the collective velocity of the system and *K* is the Luttinger liquid parameter of the system^7,8,26^. It is not possible to find the analytical expression for *K* in terms of *Δ* and *U* for the Kitaev model Hamiltonian. like many other quantum many body systems. The analytical expressions for *K* are only found for a few quantum body systems^11,26,40^. But the RG equation of *K* is related with the *Δ* and *U*. Similarly the RG equations for *Δ* and *U* are also related with the *K*. In this way *Δ*, *U* and *K* are related with each other and also evolve through the renormalization group flow. *K* determines the nature of interaction. *K* < 1 and *K* > 1 characterizes the repulsive and attractive interactions respectively, whereas *K* = 1 characterizes non-interacting case^26^. To get the correct physical picture of quantum criticality for this model Hamiltonian, RG study is essential^11^. Here we present the second (2nd) order and third (3rd) order RG equations. Detailed derivations of bosonized Hamiltonian and RG equations are relegated to the “Methods” section. The analytical expressions for the 2nd order RG are the following:

7 $$\begin{array}{ccc}\frac{d\Delta }{dl} & = & \left[2-\frac{1}{K}\left(1+\frac{\mu }{\pi \sqrt{\pi }}\right)\right]\Delta \\ \frac{dU}{dl} & = & \left(2-4K\right)U\\ \frac{dK}{dl} & = & \frac{{\Delta }^{2}}{8}-8{K}^{2}{U}^{2}.\end{array}$$

The analytical expressions for the 3rd order RG are the following:

8 $$\begin{array}{ccc}\frac{d\Delta }{dl} & = & \left[2-\frac{1}{K}\left(1+\frac{\mu }{\pi \sqrt{\pi }}\right)\right]\Delta +2K\Delta {U}^{2}\\ \frac{dU}{dl} & = & \left(2-4K\right)U\\ \frac{dK}{dl} & = & \frac{{\Delta }^{2}}{8}-8{K}^{2}{U}^{2}.\end{array}$$

We notice from these two sets of RG equations that the 2nd and 3rd order RG equations differ from each other in different analytical relations for $\frac{d\Delta }{dl}$. The origin of this extra term for the 3rd order RG equation has presented in "Methods" section explicitly. It is very clear from these sets of RG equations for interacting Kitaev model Hamiltonian that all the parameters are related with each other during the RG flow process.

When we consider *U* = 0, then 2nd order and 3rd order RG equations reduced to the same RG equations, this is nothing but the QBKT equation. Similarly when *Δ* = 0, the 2nd order and 3rd order RG equations reduce to a another RG equation for QBKT which we will discuss explicitly in the section of quantum Berezinskii-Kosterlitz-Thousless transition.

## Results Along with Physical Interpretations

**Figure 1:**
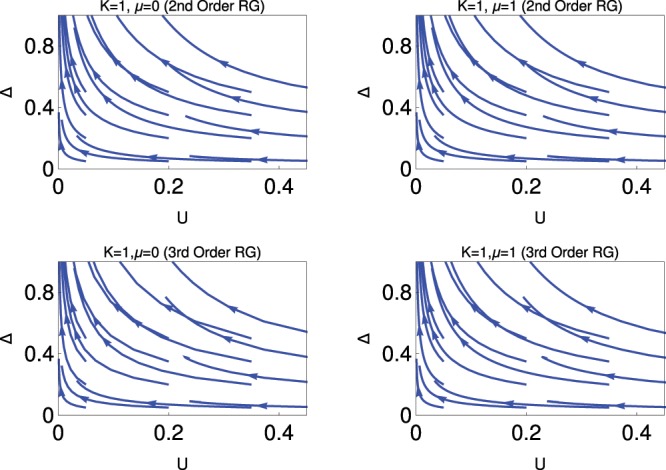
Behaviour of RG flow lines in *Δ* − *U* plane for fixed *K* = 1. The upper and lower panels are for second and third order RG study respectively, each panel consists of two figures for two different values of *μ* = 0 (left) and *μ* = 1 (right).

**Figure 2:**
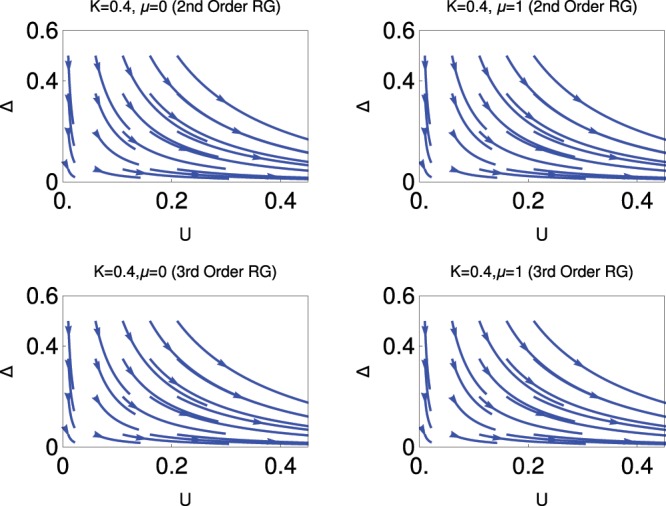
Behaviour of RG flow lines in *Δ* − *U* plane for fixed *K* = 0.4. Upper panel, 2nd order RG; lower panel, 3rd order RG; for *μ* = 0 (left) and *μ* = 1 (right).

**Figure 3:**
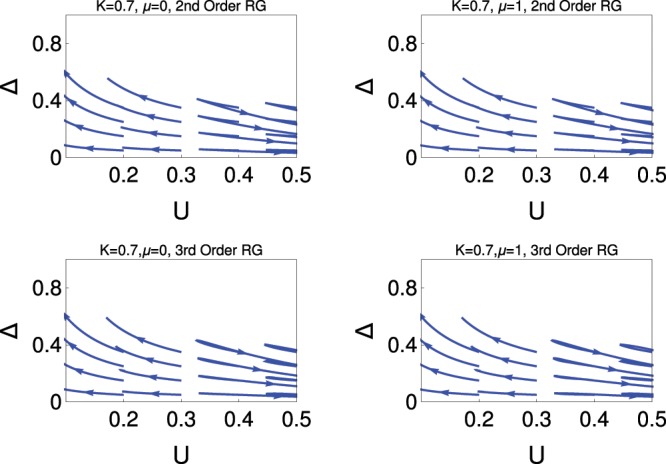
Behaviour of RG flow lines in *Δ* − *U* plane for *K* = 0.7. Upper panel, 2nd order RG; lower panel, 3rd order RG; for *μ* = 0 (left) and *μ* = 1 (right).

In this section, we present the results based on the solution of the RG Eqs.  (7) and (8). We follow the standard procedure of RG theory during the study of RG flow diagram. In this context, the reader is referred to the following references of the manuscript 7,8,11,27–29,40,44. There are two couplings *Δ* and *U*, and we present the results (Figs. 1, 2, 3) based on how these couplings behave under the RG flow equations. Finally we find different emergent quantum phases based on how the RG flow lines behave for the different values of *K*. It is customary to present the RG flow diagram in plane with the variation with two coupling constant (here *Δ* and *U*)^7,8,11,26,36–38,40–42^.

 Fig. 1 shows the behaviour of RG flow lines in the *Δ* − *U* plane for *K* = 1 (non interacting phase). The behaviour of the RG flow lines are the same for both *μ* = 0 and *μ* = 1 in 2nd order RG and 3rd order RG. For these values of *K* and *μ*, the system is always in the topological superconducting phase. It is clear from our study that the result of 3rd order RG shows more stable topological superconducting phase compared to 2nd order RG. It is also clear from the analytical expressions of 3rd order RG with an added contribution, which helps to stabilize the topological superconducting phase for the following reasons. In the 3rd order RG equation, *Δ* favours stronger coupling phase due to the extra additional contribution in the $\frac{d\Delta }{dl}$; at the same time it has also positive contribution in $\frac{dk}{dl}$; which leads *K* to the higher value during the RG flow from its initial value. We have already noticed that higher values of *K* favour the topological superconducting phase. Thus it is very clear from this study that the system always prefer to stay in the topological superconducting phase for any values of *U* for the non interacting phase.

Physical explanation for the topological superconducting phase is the following:

We observe from this figure that RG flow lines for the coupling, *Δ*, are flowing off to the strong coupling phase. This strong coupling dominated phase is nothing but the spinless p-wave superconducting phase of the Kitaev model Hamiltonian which induces the topological superconducting phase with gapless Majorana edge modes in the system. For non-topological (conventional) superconducting phase there are no gapless edge modes. It is very clear from the analysis of Eq.  (4), that there are no Majorana fermions at the two end points of that lattice Hamiltonian, this study is for *U* = 0. But we predict the existence of topological superconducting phase for finite *U* in this RG study for different values of *μ* for *K* = 1, as it effectively system behave as a non-interacting system.

It is very clear from this study that the system always prefer to stay in the topological superconducting phase for any values of *U* for *K* = 1. This is the further advancement of results beyond the existence results of Kitaev model Hamiltonian.

 Fig. 2 shows the RG flow diagram with *K* = 0.4, system is in the more correlated phase, for *μ* = 0 and *μ* = 1. We observe that the system is always in the CDW insulating phase. The results are the same for the 2nd order and 3rd order RG study. This is because there is no additional contribution to the third order RG equation from the sine-Gordon coupling term *U*. This term is less relevant for higher order compared to the sine-Gordon coupling term of *Δ*. It is very clear from this figure that the system always prefer to stay in the CDW phase for any values of *Δ* for *K* = 0.4 as if effectively there is no existence of superconducting pairing.

Now we explain the results physically, which we obtain from the study of fig. 1 and Fig. 2. These results obtained from the study of Fig. 1 and Fig. 2 are physically consistent for two different regions of *K*. In one regime, i.e., for the higher values of *K*, the sine-Gordon coupling term for *Δ* is more relevant and for the other regime, i.e., for smaller values of *K*, the sine-Gordon coupling term for *U* is more relevant compared to the sine-Gordon coupling term for *Δ*. For this situation, one can ignore the kinetic energy term. We have already discussed the mean field aspect of CDW phase in the previous section.

Thus it is clear from the study of these two figures that there is no topological quantum phase transition from the topological superconducting phase to the CDW phase for these values of *K*. To get the topological quantum phase transition, we must consider other values of *K*, which are lying between these two values *K*, as we notice in Fig. 3.

 Fig. 3, shows the results when *K* = 0.7. This study shows very interesting behaviour for topological quantum phase transition which depends on the strong repulsive interactions. It reveals that for smaller values of repulsive interactions (*U*), RG flow lines for the coupling *Δ* are flowing off to the strong coupling phase, as we already discuss this phase correspondence to the topological superconducting phase. But for higher values of *U*, RG flow lines are flowing off to the strong coupling phase of *U*, i.e., the system is dominated by the CDW phase. For this value of *K*, the coupling *Δ* and *U* are competing with each other to finalize the topological quantum phase transition. This transition is the topological phase transition. The system can be characterized by the topological invariant quantity for the topological superconducting phase but there is no topological invariant quantity to characterize the CDW insulating state. The study of 3rd order RG reveals that the topological quantum phase transition occurs for the higher value of *U*, i.e., the system prefers to stay more in topological superconducting phase. This behaviour of topological superconducting phase is consistent with the study of Fig. 1. In this study, the parameters (*t*, *μ*, *Δ*, U), of the model Hamiltonian are in arbitrary units. One may consider it to be in the scale of meV.

## A Comparison of Results Between the First Three Figures

It is clear from Fig. 1 and Fig. 2 that one can interpret the system effectively as a single phase system, i.e., topological superconducting phase or CDW phase for these regions of parameter space from our RG study depending on the value of *K*. There is no evidence of Majorana-Ising transition. In Fig. 3, we observe the topological superconducting phase to the CDW phase transition. This is an example of topological quantum transition for the following reasons. Our model Hamiltonian consists of two sine-Gordon coupling terms of the dual field that stabilize competing orders and allows different types of quantum phase transition. The system can be characterized by the topological invariant quantity for the topological superconducting phase but there is no topological invariant quantity to characterize the insulating state.

## A Study of Quantum Berezinskii-Kosterlitz-Thouless Transition

The QBKT physics with the topological back ground has not between studied for the Hamiltonian *H* (Eq. 5 and Eq. 6). Here we reduce our model Hamiltonian into two QBKT Hamiltonians in different regimes of parameter space. There is only one sine-Gordon coupling term for each Hamiltonian. Therefore one cannot neglect the kinetic energy contribution of the Hamiltonian. The sine-Gordon coupling term gives the confining potential and the *H*_0_ part of the Hamiltonian gives the kinetic energy contribution, or in other words gives the quadratic fluctuations of the *ϕ* and *θ* fields. Therefore the competition between these fluctuations of these fields and confining potential sine-Gordon coupling term finally selects the stabilize quantum phase of the system, instead of competition between the two sine-Gordon coupling terms of the total RG equation. We have already discussed that the BKT transition is topological in nature and the basic aspect of QBKT. Earlier literature has mostly studied classical BKT. In QBKT, there is no topological transition dependent on the temperature as we observe for classical BKT. For this case topological transition depends on the strength of sine-Gordon coupling and the Luttinger liquid parameter.

In this section, we show explicitly how we derive the QBKT equations from the whole RG equations to show that the physics of QBKT is inherent for this model Hamiltonian system. We have presented the results of RG flow lines (*Δ* with *U*) in the previous section. We extract two different kinds of quantum phases, one topological and the other non-topological. Here we study how the RG flow lines behave for *Δ* with *K* and also *U* with *K*. At the same time, we ask whether there is any possibility of finding any new emergent quantum phases that we have not found from the study of the whole set of RG equations.

The different limits of the total RG equations (Eq.  (7) and Eq.  (8)) yield two QBKT equations, the first one is for *U* = 0,

9 $$\begin{array}{ccc}\frac{d\Delta }{dl} & = & \left[2-\frac{1}{K}\left(1+\frac{\mu }{\pi \sqrt{\pi }}\right)\right]\Delta \\  &  & \frac{dK}{dl}=\frac{{\Delta }^{2}}{8}.\end{array}$$

The second set of quantum RG equations can be obtained from the consideration *Δ* = 0:

10 $$\begin{array}{ccc}\frac{dU}{dl} & = & \left(2-4K\right)U\\ \frac{dK}{dl} & = & -8{K}^{2}{U}^{2}.\end{array}$$

This is the standard format of the BKT equations. We solve these two equations exactly and study the RG flow diagram. Here we present the length-scale dependent study to get further insight into the RG results and at the same time to unveil the physics of asymptotic-freedom like behaviour for this model Hamiltonian system. To the best of our knowledge, this is the first study of QBKT for the interacting topological state of matter and also asymptotic-freedom like behaviour for the interacting topological system. At the same time physics of asymptotic-freedom like behaviour is very rare in quantum many body condensed matter system^7,8,35,37^.

## Alternative Derivation of QBKT Equations

One can also obtain the same set of RG equations from a different consideration, i.e., starting from the two different model Hamiltonians *H*_1_ (Eq. 6 for *U* = 0) and *H*_2_ (Eq. 6 for *Δ* = 0):

11 $${H}_{1}={H}_{0}+\frac{\Delta }{2}\int cos\left(2\sqrt{\frac{\pi }{K}}\theta (x)\right)dx-\mu \sqrt{\frac{K}{\pi }}\int ({\partial }_{x}\varphi (x))dx,$$

12 $${H}_{2}={H}_{0}+U\int cos(4\sqrt{\pi K}\varphi (x))dx-\mu \sqrt{\frac{K}{\pi }}\int ({\partial }_{x}\varphi (x))dx.$$

Starting from these two Hamiltonians, following the analytical scheme that we have proposed in the “Methods” section, one can derive the two sets of RG equations, which are the same as Eqs. (9) and (10).

One can do the exact solution of these RG equations. Detailed derivation is relegated to the “Methods” section; here, we only present the final forms

13 $$\Delta =\sqrt{{{\Delta }_{0}}^{2}+32(K-{K}_{0})-16ln(K/{K}_{0})(1+\frac{\mu }{\pi \sqrt{\pi }})}.$$

14 $$U=\sqrt{{{U}_{0}}^{2}+\frac{1}{2}(1/K-1/{K}_{0})+ln(K/{K}_{0})}.$$

Here *Δ*_0_, *K*_0_ and *U*_0_ are the initial values of *Δ*, *K* and *U* respectively. Now, we present the results based on exact calculation of QBKT (Eq. 13 and Eq. 14). We also notice that the QBKT equations are the same for 2nd order and 3rd order RG.

The analytical expression for the first set of RG equations (Eq.  (9)) contains *μ* but the second set of RG equations (Eq.  (10)) does not contain *μ* for the following reason. The first set of equations is for the *θ*(*x*) field but the field associated with the chemical potential is the *ϕ*(*x*) field. These two fields are dual to each other, therefore one cannot absorb one field to another by a shift of chemical potential for this Hamiltonian (*H*_1_) as one can do for the Hamiltonian *H*_2_.

## Results of QBKT

**Figure 4:**
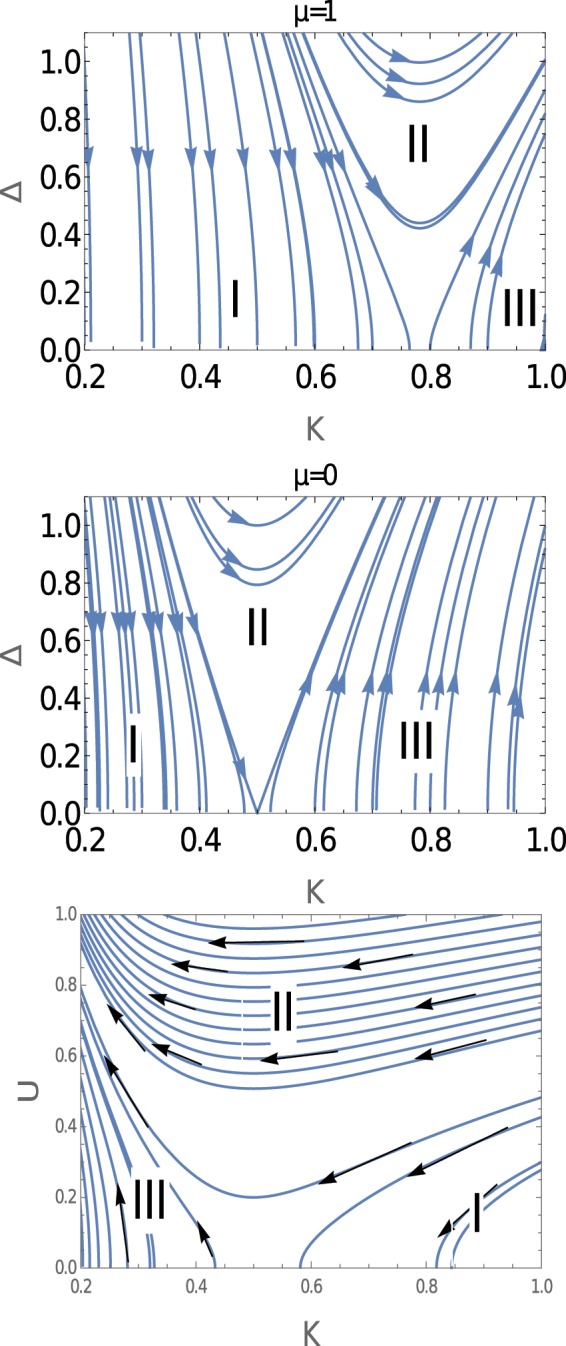
RG flow behaviour based on exact solutions (Eq. 13 and Eq. 14). Top and middle, *Δ* with *K*; lower, U with K. The region I, II, III are explained in text.

In Fig. 4, we present the result based on the exact solutions (Eq. 13 and Eq. 14) of RG equations for different values of chemical potential. This figure consists of three panels. The upper and middle panels present the results for *Δ* with *K* for *μ* = 1 and *μ* = 0 respectively. We observe the existence of three regions: gapless Luttinger liquid region, topological superconducting region, and a crossover region. The most important result of this study is that as we decrease the value of chemical potential the Luttinger liquid regime (I), i.e., the gapless regime, decreases and as a consequence the topological superconducting phase region (III) and the crossover region (II) increases. The lower panel of Fig. 4 shows the result for *U* with *K*. It also contains different regimes of phases. The common phase for these two QBKT is the gapless Luttinger liquid phase. In the strong coupling limit, the system is in the CDW phase. This phase has also crossover phase but the direction is opposite with *k*, i.e., the system drives from gapless Luttinger liquid phase to CDW phase.

For these two QBKT, there is no transition from topological superconducting phase to non-topological CDW phase. This is because the BKT Hamiltonian only contains a single sine-Gordon coupling term.

### Length-scale Dependence Study: Asymptotic Freedom Like Behaviour

RG theory is a formalism that relates the physics at different length-scales in condensed matter physics and the physics at different energy-scales in high-energy physics^35–37^.

In the present study, we show explicitly how the physics of topology appears at different length-scales of the system and also obtain the signature of asymptotic freedom like behaviour and finally the effect of interaction on it. The authors of ref. ^43^ and ref. ^44^ have shown that in QCD, the gauge theory of quarks and gluons are asymptotically free, i.e., the coupling vanishes at very short distance (large momentum) and grows at large distance (small momentum). This allowed us to understand why quarks seemed free inside the nucleons in deep inelastic scattering and are confined at large distance.

But the present problem is not QCD. Here we solve a topological system in presence of interaction. At the same time we are not interpreting our results in terms of quark and gluon physics, rather in terms of topological quantum phase transition, therefore we should interpret our results from the length-scale dependent asymptotic freedom like behaviour for the interacting topological system.

One of the successful part of the RG theory is the observation of asymptotic freedom for high-energy physics and the other is the BKT physics for condensed matter physics, i.e, the two extreme ends of theoretical physics. In this study we are successful in unifying these two observations of RG theory in a single framework of topological state of matter.

Length-scale dependence study brings out the concept of asymptotic freedom like behaviour in the RG flow sense. This asymptotic freedom is a feature of all RG flows with a marginally relevant perturbation. We would like to explain it explicitly through the *β* function explanation. This can be written as ${\beta }_{\lambda }=\frac{d\lambda }{dlnL}=C{\lambda }^{2}$ (here *β*_*λ*_ is the *β* function of the RG theory from where one can predict the nature of the RG flow lines of coupling constant *λ*; in quantum field theory flow lines are defined in energy scale but here we define RG flow lines in length scale). Here *λ* is the coupling constant and *C* > 0 is a constant. As a result, the effective coupling is weak at short distance and strong at long distances.

**Figure 5:**
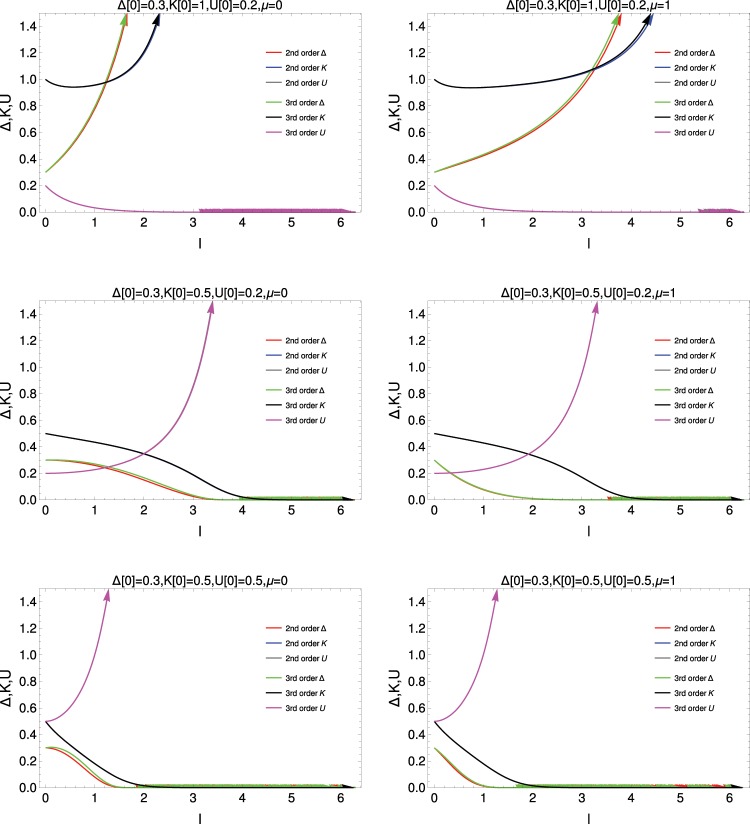
Variation of *Δ*, *U* and *K* with length scale in second order and third order RG studies. *Δ*(0) = 0.3 is the same in all three panels; *μ* = 0 at left and *μ* = 1 at right; *K*(0) = 1 in the upper panel, *K*(0) = 0.5 in middle and lower panels; *U*(0) = 0.2 in upper and middle panels; *U*(0) = 0.5 in lower panel.

In Fig. 5, we present the length scale dependence study of the 2nd order and 3rd order RG equations (Eq.  (7) and Eq.  (8)). The upper panel of the figure shows that both 2nd order and 3rd order RG equations present the same variation of *Δ*, *K*, *U* with length scale. *K*, *Δ* increase with length scale and *U* decreases as length scale increases. It implies that the system is in the topological superconducting state. The same behaviour is seen for two different values of *μ*. The middle panel shows interesting behaviour for the lower value of *K*(0) = 0.5. The decaying rate for *Δ* is higher for *μ* = 1. The system is in the insulating CDW phase and there is no topological invariant quantity to describe this phase.

The lower panel, with higher value of interaction strength (*U*(0) = 0.5) for *K*(0) = 0.5, i.e., the system is in the more strongly correlated regime than in the upper and middle panels, reveals that *U* rises very sharply with length scale and *Δ* and *K* decrease rapidly with length scale. Thus the system is in the CDW phase.

**Figure 6:**
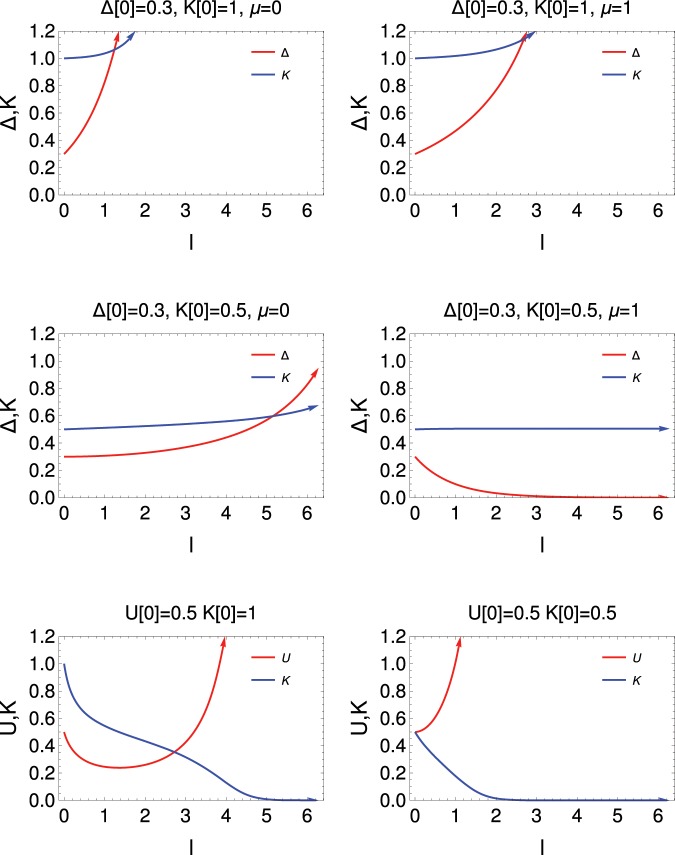
Length scale dependence in QBKT. Upper and middle panels show variation of *Δ* and *K* with length scale with *Δ* = 0.3; upper, *K*(0) = 1; middle *K*(0) = 0.5; *μ* = 0 at left and *μ* = 1 at right. Lower panel shows variation of *U* and *K* with length scale with *U*(0) = 0.5: left, *K*(0) = 1; right, *K*(0) = 0.5.

In Fig. 6, we present the length-scale dependence study for QBKT for both sets of RG equations (Eq.  (9) and Eq.  (10)). This figure shows that when *K*(0) = 1 (upper panel), the system shows asymptotic freedom like behaviour with increase of *Δ* with length-scale for two different values of *μ*, whereas there is no signature of asymptotic behaviour for the smaller value of *K*(0) = 0.5 (middle panel). The lower panel shows the variation of *U* and *K* with length-scale for two different values of *K*(0) = 1 and *K*(0) = 0.5. It also shows the asymptotic freedom like behaviour for the coupling *U*, this behaviour is more sharper for *K*(0) = 0.5.

In the lower panel, there is no *μ* dependent result because the RG equation for *U* coupling does not depend of *μ*. It is clear from the results in the upper and middle panels that the effect of *K* is quite pronounced on the asymptotic freedom like behaviour. We notice that lower values of *K* wash out the asymptotic freedom like behaviour of *Δ*. But its effect on *U* is just the opposite: it promotes the asymptotic freedom like behaviour very sharply.

## A Comparison Between the Length-scale Dependence Studies of Total RG Equations and QBKT Equations

The results in Fig. 5 are based on total RG equations (Eq.  (7) and Eq.  (8)), i.e, when two sine-Gordon coupling terms are present that are competing with each other. But in Fig. 6, QBKT contains only one coupling term for each set of QBKT RG equations, therefore there is no competition between two sine-Gordon coupling terms. But the most important feature is that we still get the asymptotic freedom like behaviour in both Fig. 5 and Fig. 6.

The properties of the two different asymptotic freedom regions are different. For the first one the system can be described in terms of topological invariant number with bulk gap and gapless edge modes. But for the second case of higher values of repulsive interaction, there is no topological invariant number to characterize the system, the asymptotic freedom like region is gapped, and there is no gapless edge mode. We do rigorous quantum field theoretical renormalization group calculation to solve this problem. There are quite a few studies in the literature^17–24,41,42,45,46^ of interacting topological state but none has shown these new and important results.

Apart from the topological superconducting and CDW phase, we have found asymptotic freedom like and also gapless LL phase in this study, as a emergent phase in the system which is absent in the literature.

## Discussion

We have presented results of interacting Kitaev model for different regimes of the parameter space. We have shown appearence of two new emergent phases, one is gapless Luttinger liquid phase and other is asymptotie freedom like behaviour. Apart from that we have also found conventional topological superconducting phase and CDW phase. We have also obtained exact solutions for the two sets of QBKT renormalization group equations. We have also presented quite a few new and important results on length -scale dependent study to unveil the underlying topology. In this study, we have unified the physics of QBKT and asymptotic freedom for interacting topological state of matter. This work provides a new perspective on the study of topological state of interacting quantum matter and also for QBKT.

## Methods

### A derivation of bosonized Hamiltonian

Here we present a detail derivation of the bosonized Hamiltonian (Eq. 6) from the Kitaev model Hamiltonian (Eq.  (1)). At first we present the Kitaev model Hamiltonian in presence of repulsive interaction in spinless fermion representation (Eq.  (1)).

15 $$\begin{array}{ccc}H & = & -t{\sum }_{i=1}^{N-1}({{c}_{i}}^{\dagger }{c}_{i+1}+h.c)+\Delta {\sum }_{i=1}^{N-1}({c}_{i}{c}_{i+1}+h.c)\\  &  & +U{\sum }_{i=1}^{N-1}(2{{c}_{i}}^{\dagger }{c}_{i}-1)(2{{c}_{i+1}}^{\dagger }{c}_{i+1}-1)-\mu {\sum }_{i}^{N}{{c}_{i}}^{\dagger }{c}_{i}.\end{array}$$

We recast this model Hamiltonian in terms of spin-1/2 operators by using the Jordan-Wigner transformation to connect the spinless fermion operators to the spin-1/2 operator, which is below.

$${{c}_{j}}^{\dagger }=({{s}_{j}}^{+}){\Pi }_{l=1}^{j-1}({-{s}_{l}}^{z}).{c}_{j}=({{s}_{j}}^{-}){\Pi }_{l=1}^{j-1}({-{s}_{l}}^{z})$$

After this transformation the Kitaev Hamiltonian become

16 $$H={\sum }_{n}[(\frac{t+\Delta }{2}){S}_{n}^{x}{S}_{n+1}^{x}+\frac{(t-\Delta )}{2}{S}_{n}^{y}{S}_{n+1}^{y}+U{S}_{n}^{z}{S}_{n+1}^{z}-\mu {S}_{n}^{z}].$$

The above two Hamiltonians are free from *K*. Therefore, now our main task is to find the analytical expression for spin-1/2 operators in terms of in terms of bosonized fields *ϕ* and *θ* and that also show how *K* appears in the Kitaev model.

We present spin operators interms of *ϕ*, *θ* and *K*^26^,

$$\begin{array}{ccc}{{S}_{n}}^{+} & = & {S}_{n}(x)+i{S}_{n}(y)={e}^{-i\sqrt{\frac{\pi }{K}}\theta (x)}[{(-1)}^{x}+cos(2\sqrt{\pi K}\varphi (x)]\\ {{S}_{n}}^{-} & = & {S}_{n}(x)-i{S}_{n}(y)={e}^{i\sqrt{\frac{\pi }{K}}\theta (x)}[{(-1)}^{x}+cos\left(2\sqrt{\pi K}\varphi (x)\right.]\\ {{S}_{n}}^{x} & = & [cos(2\sqrt{\pi K}\varphi (x))+{(-1)}^{n}]cos\left(\sqrt{\frac{\pi }{K}}\theta (x)\right.\\ {{S}_{n}}^{y} & = & -[cos(2\sqrt{\pi K}\varphi (x))+{(-1)}^{n}]sin\left(\sqrt{\frac{\pi }{K}}\theta (x)\right.\\ {{S}_{n}}^{z} & = & {(-1)}^{n}cos(2\sqrt{\pi K}\varphi (x))+\sqrt{\frac{K}{\pi }}{\partial }_{x}\varphi (x)\end{array}$$

Thus finally we obtain the bosonized version of Kitaev model Hamiltonian by using the analytical expressions of quantum spin operators.

17 $$H={H}_{0}+\frac{\Delta }{2}\int cos\left(2\sqrt{\frac{\pi }{K}}\theta (x)\right)dx+U\int cos(4\sqrt{\pi K}\varphi (x))dx-\mu \sqrt{\frac{K}{\pi }}\int ({\partial }_{x}\varphi (x))dx,$$

${H}_{0}=\frac{v}{2}\int [{({\partial }_{x}\theta )}^{2}+{({\partial }_{x}\varphi )}^{2}]dx$. We notice that *H*_0_ appears with out *K*, therefore the rest three terms of Eq. 17 appear as a function of *K* otherwise it appears with out *K*.

### Cumulant expansion

The cumulant-generating function (*Θ*(*k*)) is the logarithm of the characteristic function (*Φ*(*k*)). Characteristic function is defined as,

18 $$\begin{array}{ccl}\Phi (k) & = & \left\langle {e}^{ikA}\right\rangle ={\sum }_{r=0}^{\infty }\frac{{(ik)}^{r}}{r!}\left\langle {A}^{r}\right\rangle \\  & = & 1+(ik)\left\langle A\right\rangle +\frac{{(ik)}^{2}}{2!}\left\langle {A}^{2}\right\rangle +\frac{{(ik)}^{3}}{3!}\left\langle {A}^{3}\right\rangle +\ldots .\end{array}$$

where *A* is a random variable and the Taylor expansion of the exponential function is for small values of *k*. Characteristic function and the moment-generating function (*M*_*A*_(*k*)) are related since ${M}_{A}(k)=\left\langle {e}^{kA}\right\rangle$. Thus we have the relation *Φ*(−*ik*) = *M*_*A*_(*k*) with $\left\langle {A}^{r}\right\rangle$ as moments of random variable *A*. These moments can be calculated by differentiation of the expansion of the exponential function with respect to *k*. By taking the logarithm of *Φ*(*k*) we get the expansion of *Θ*(*k*) in terms of cumulants (*Γ*_*r*_) as,

19 $$\begin{array}{lll}\Theta (k) & = & log\Phi (k)\\  & = & log\left[1+(ik)\left\langle A\right\rangle +\frac{{(ik)}^{2}}{2!}\left\langle {A}^{2}\right\rangle +\frac{{(ik)}^{3}}{3!}\left\langle {A}^{3}\right\rangle +\ldots .\right].\end{array}$$

Thus Eq.  (15) becomes

20 $$\begin{array}{ccc}\Theta (k) & = & \left((ik)\left\langle A\right\rangle +\frac{{(ik)}^{2}}{2!}\left\langle {A}^{2}\right\rangle +\frac{{(ik)}^{3}}{3!}\left\langle {A}^{3}\right\rangle +\ldots .\right)-\frac{{\left((ik)\left\langle A\right\rangle +\frac{{(ik)}^{2}}{2!}\left\langle {A}^{2}\right\rangle +\frac{{(ik)}^{3}}{3!}\left\langle {A}^{3}\right\rangle +\ldots .\right)}^{2}}{2}\\  &  & +\frac{{\left((ik)\left\langle A\right\rangle +\frac{{(ik)}^{2}}{2!}\left\langle {A}^{2}\right\rangle +\frac{{(ik)}^{3}}{3!}\left\langle {A}^{3}\right\rangle +\ldots .\right)}^{3}}{3}\end{array}$$

Finally, after the log expansion we obtain $\Theta (k)=(ik){\Gamma }_{1}+\frac{{(ik)}^{2}}{2}{\Gamma }_{2}+\frac{{(ik)}^{3}}{6}{\Gamma }_{3}+\ldots ..$. Thus we have, up to third order, ${\Gamma }_{1}=\left\langle A\right\rangle$, ${\Gamma }_{2}=\left\langle {A}^{2}\right\rangle -{\left\langle A\right\rangle }^{2}$, ${\Gamma }_{3}=\left\langle {A}^{3}\right\rangle -3\left\langle {A}^{2}\right\rangle \left\langle A\right\rangle +2{\left\langle A\right\rangle }^{3}$. While calculating the average $\left\langle {e}^{\Omega }\right\rangle$ we can use the cumulant expansion.

21 $$\left\langle {e}^{\Omega }\right\rangle ={\Phi }_{\Omega }(-i)={e}^{ln{\Phi }_{\Omega }(-i)}={e}^{{\Theta }_{\Omega }(-i)}={e}^{\left({\sum }_{r=0}^{\infty }\frac{1}{r!}{\Gamma }_{r}\right)}={e}^{\left\langle \Omega \right\rangle +\frac{1}{2}(\left\langle {\Omega }^{2}\right\rangle -{\left\langle \Omega \right\rangle }^{2})+\ldots }$$

### Derivation of exact solutions for the quantum BKT RG flow lines for *Δ* and *U*

We may write the equation for $\frac{d\Delta }{dK}$ from Eq.  (9) as

$$\frac{d\Delta }{dK}=\frac{8[2-\frac{1}{K}(1+\frac{\mu }{\pi \sqrt{\pi }})]}{\Delta }$$

We can write the integration constant as

$$C=\frac{1}{2}{\Delta }^{2}-16K+8(1+\frac{\mu }{\sqrt{\pi }})ln(K)$$

One can evaluate the constant from the initial value of *Δ* and *K*, i.e., *Δ*_0_ and *K*_0_.

$$\Delta =\sqrt{{{\Delta }_{0}}^{2}+32(K-{K}_{0})-16(1+\frac{\mu }{\pi \sqrt{\pi }})ln(K/{K}_{0})}$$

Similarly one can find the exact solution for the flow lines for *U* as

$$U=\sqrt{{{U}_{0}}^{2}+ln(K-{K}_{0})+\frac{1}{2}(1/K-1/{K}_{0})}$$

Finally we obtain the exact solution for RG flow lines by integrating the RG equations.

### Derivation of renormalization group equations

Our starting point is the bosonized Hamiltonian,

$${H}_{XYZ}={H}_{0}+a\int cos\left(2\sqrt{\frac{\pi }{K}}\theta (x)\right)dx+U\int cos(4\sqrt{\pi K}\varphi (x))dx-\mu \sqrt{\frac{K}{\pi }}\int ({\partial }_{x}\varphi (x))dx$$

where ${H}_{0}=\frac{v}{2}\int [{({\partial }_{x}\theta )}^{2}+{({\partial }_{x}\varphi )}^{2}]dx$, where *v* is the collective velocity of this one dimensional model Hamiltonian system^7,8,41^. Here, we consider *Δ* = 2*a* for the smoothness of calculation, but we finally present the RG equations in terms of *Δ*. Now we write the partition function ${\mathcal{Z}}$ in terms of fields as,

22 $${\mathcal{Z}}=\int {\mathcal{D}}\varphi {\mathcal{D}}\theta {e}^{-{S}_{E}[\varphi ,\theta ]},$$

where *S*_*E*_ is the Euclidean action which can be written as ${S}_{E}=-\int dr{\mathcal{L}}=-\int dr({{\mathcal{L}}}_{0}+{{\mathcal{L}}}_{int})$, where *r* = (*τ*, *x*). Thus the partition function is given by

23 $$\begin{array}{ccc}{\mathcal{Z}} & = & \int {\mathcal{D}}\varphi {\mathcal{D}}\theta exp\left[-{\int }_{-\Lambda }^{\Lambda }\frac{d\omega }{2\pi }| \omega | \left(\frac{| \varphi (\omega ){| }^{2}}{2K}+\frac{K| \theta (\omega ){| }^{2}}{2}\right)\right.\\  &  & \,-\left.\int dr\left(\frac{i\mu \sqrt{\pi }}{v}({\partial }_{r}\theta )+Ucos(4\sqrt{\pi }\varphi )+acos(2\sqrt{\pi }\theta )\right)\right].\end{array}$$

The first and second terms of the exponent of the above equation are ${{\mathcal{L}}}_{0}$ and ${{\mathcal{L}}}_{int}$ respectively.

Now we divide the fields into slow and fast modes and integrate out the fast modes. The field *ϕ* is *ϕ*(*r*) = *ϕ*_*s*_(*r*) + *ϕ*_*f*_(*r*) similarly the *θ* is *θ*(*r*) = *θ*_*s*_(*r*) + *θ*_*f*_(*r*), where

24 $${\varphi }_{s}(r)={\int }_{-\Lambda /b}^{\Lambda /b}\frac{d\omega }{2\pi }{e}^{-i\omega r}\varphi (\omega )\ \ \ and\ \ \ \ {\varphi }_{f}(r)={\int }_{\Lambda /b < | {\omega }_{n}|  < \Lambda }\frac{d\omega }{2\pi }{e}^{-i\omega r}\varphi (\omega ),$$

25 $${\theta }_{s}(r)={\int }_{-\Lambda /b}^{\Lambda /b}\frac{d\omega }{2\pi }{e}^{-i\omega r}\theta (\omega )\ \ \ and\ \ \ \ {\theta }_{f}(r)={\int }_{\Lambda /b < | {\omega }_{n}|  < \Lambda }\frac{d\omega }{2\pi }{e}^{-i\omega r}\theta (\omega ).$$

Here *Λ* is the cut-off to start with and *b* is a factor greater than one. It is clear from the above definitions of faster and slower mode. One can make the average over the fast mode in order to get an effective action for the slower mode. Thus ${\mathcal{Z}}$ is

26 $${\mathcal{Z}}=\int {\mathcal{D}}{\varphi }_{s}{\mathcal{D}}{\varphi }_{f}{\mathcal{D}}{\theta }_{s}{\mathcal{D}}{\theta }_{f}{e}^{-{S}_{s}({\varphi }_{s},{\theta }_{s})}{e}^{-{S}_{f}({\varphi }_{f},{\theta }_{f})}{e}^{-{S}_{int}(\varphi ,\theta )}.$$

Using the relation ${\left\langle A\right\rangle }_{f}=\int {\mathcal{D}}{\varphi }_{f}{e}^{-{S}_{f}({\varphi }_{f},{\theta }_{f})}A$, one can write

27 $${\mathcal{Z}}=\int {\mathcal{D}}{\varphi }_{s}{\mathcal{D}}{\theta }_{s}{e}^{-{S}_{s}({\varphi }_{s},{\theta }_{s})}{\left\langle {e}^{-{S}_{int}(\varphi ,\theta )}\right\rangle }_{f}.$$

We write the effective action as

28 $${e}^{-{S}_{eff}({\varphi }_{s},{\theta }_{s})}={e}^{-{S}_{s}({\varphi }_{s},{\theta }_{s})}{\left\langle {e}^{-{S}_{int}(\varphi ,\theta )}\right\rangle }_{f}.$$

Taking ln on both sides give, ${S}_{eff}({\varphi }_{s},{\theta }_{s})={S}_{s}({\varphi }_{s},{\theta }_{s})-ln{\left\langle {e}^{-{S}_{int}(\varphi ,\theta )}\right\rangle }_{f}$. By writing the cumulant expansion up to third order, we have

29 $$\begin{array}{ccc}{S}_{eff}({\varphi }_{s},{\theta }_{s}) & = & {S}_{s}({\varphi }_{s},{\theta }_{s})+{\left\langle {S}_{int}(\varphi ,\theta )\right\rangle }_{f}-\frac{1}{2}\left({\left\langle {S}_{int}^{2}(\varphi ,\theta )\right\rangle }_{f}-{\left\langle {S}_{int}(\varphi ,\theta )\right\rangle }_{f}^{2}\right)\\  &  & \,+\frac{1}{6}\left({\left\langle {S}_{int}^{3}(\varphi ,\theta )\right\rangle }_{f}-3{\left\langle {S}_{int}^{2}(\varphi ,\theta )\right\rangle }_{f}{\left\langle {S}_{int}(\varphi ,\theta )\right\rangle }_{f}+2{\left\langle {S}_{int}(\varphi ,\theta )\right\rangle }_{f}^{3}\right).\end{array}$$

At first we derive the 2nd order RG equations for *μ* = 0 and then we extend it for finite *μ*.

30 $$\left\langle {S}_{int}(\theta ,\varphi )\right\rangle =\int dr\left[a{\left\langle cos\left(2\sqrt{\pi }\theta (r)\right)\right\rangle }_{f}\right]+\int dr\left[U{\left\langle cos\left(4\sqrt{\pi }\varphi (r)\right)\right\rangle }_{f}\right]$$

31


Similarly,


32


Now we calculate the second order cumulant term of the action *S*_*eff*_(*θ*, *ϕ*) (Eq. 25).

33 $$-\frac{1}{2}(\left\langle {S}_{int}^{2}\right\rangle -{\left\langle {S}_{int}\right\rangle }^{2})=-\,\frac{1}{2}\int drd{r}^{{}^{^{\prime} }}\{{a}^{2}[\ldots .]+{U}^{2}[\ldots .]+aU[\ldots .]+Ua[\ldots .]\},$$

where the dotted term represents the expectation value of the correlation function of sine-Gordon operators, which we evaluate below.

34 $$\begin{array}{ccc} &  & -\frac{{a}^{2}}{2}\int drdr^{\prime} \left\{\left\langle cos[2\sqrt{\pi }\theta (r)]cos[2\sqrt{\pi }\theta (r^{\prime} )]\right\rangle -\left\langle cos[2\sqrt{\pi }\theta (r)]\right\rangle \left\langle cos[2\sqrt{\pi }\theta (r^{\prime} )]\right\rangle \right\}\\  &  & \,=\,\frac{{a}^{2}}{4}(1-{b}^{-\frac{2}{K}})\int dr{({\partial }_{r}{\theta }_{s}(r))}^{2}.\end{array}$$

Similarly,

35 $$\begin{array}{ccc} &  & -\frac{{U}^{2}}{2}\int drd{r}^{{}^{^{\prime} }}\{\langle cos[4\sqrt{\pi }\varphi (r)]cos[4\sqrt{\pi }\varphi ({r}^{{}^{^{\prime} }})]\rangle -\langle cos\ [4\sqrt{\pi }\varphi (r)]\rangle \langle cos[4\sqrt{\pi }\varphi ({r}^{{}^{^{\prime} }})]\rangle \}\\  &  & ={U}^{2}\left(1-{b}^{-8K}\right)\int dr{({\partial }_{r}{\varphi }_{s}(r))}^{2}.\end{array}$$

We obtain the following relation by comparison of rescaled *a* term (Eq. 31) using the rescaled relation as *b* = *e*^*dl*^.

$$\bar{a}=a{b}^{(2-\frac{1}{K})}$$

Finally we write the RG equation in terms of *Δ*( = 2*a*) as, We obtain, by comparison of rescaled *U* term (Eq. 32),

$$\bar{U}=U{b}^{(2-4K)}$$

Comparison of rescaled *K* terms from the contribution of *ϕ* (Eq. 31), gives,

$$\frac{1}{\bar{K}}=\frac{1}{K}+\frac{{U}^{2}}{K}\left({b}^{2}-{b}^{(2-8K)}\right)$$

Comparison of rescaled *K* terms from the contribution of *θ* (Eq. 31), gives,

$$\bar{K}=K+\frac{K{a}^{2}}{4}\left({b}^{2}-{b}^{(2-\frac{2}{K})}\right)$$

We obtain the final form of $\frac{dk}{dl}$ after the combination of the above two contribution from *ϕ* and *θ* for *K*.Now we calculate the third order terms of cumulant expansion for the effective action (Eq. 25),

$$\begin{array}{rcl} &  & \frac{1}{6}(\left\langle {S}_{int}^{3}\right\rangle -3\left\langle {S}_{int}^{2}\right\rangle \left\langle {S}_{int}\right\rangle +2{\left\langle {S}_{int}\right\rangle }^{3})=\frac{1}{6}\int d\tau d{\tau }^{^{\prime} }d{\tau }^{^{\prime\prime} }\\  &  & \hspace{4.99878pt}\{{a}^{3}\left[\ldots ..\right]+{U}^{3}\left[\ldots ..\right]+{a}^{2}U\left[\ldots ..\right]+a{U}^{2}\left[\ldots ..\right]\}\end{array}$$

36 $$\begin{array}{c}\frac{{a}^{3}}{6}\int d\tau d{\tau }^{{\rm{^{\prime} }}}d{\tau }^{{\rm{^{\prime} }}{\rm{^{\prime} }}}[\langle cos[\sqrt{4\pi }\theta (\tau )]cos[\sqrt{4\pi }\theta ({\tau }^{{\rm{^{\prime} }}})]cos[\sqrt{4\pi }\theta ({\tau }^{{\rm{^{\prime} }}{\rm{^{\prime} }}})]\rangle \\ -\,3\langle cos[\sqrt{4\pi }\theta (\tau )]cos[\sqrt{4\pi }\theta ({\tau }^{{\rm{^{\prime} }}})]\rangle \langle cos[\sqrt{4\pi }\theta ({\tau }^{{\rm{^{\prime} }}{\rm{^{\prime} }}})]\rangle \\ \,\,\,\,\,+\,2\langle cos[\sqrt{4\pi }\theta (\tau )]\rangle \langle cos[\sqrt{4\pi }\theta ({\tau }^{{\rm{^{\prime} }}})]\rangle \langle cos[\sqrt{4\pi }\theta ({\tau }^{{\rm{^{\prime} }}{\rm{^{\prime} }}})]\rangle ]\\ \,\,\,\,\,\,\,\,\,=\,(\frac{{a}^{3}}{24})(6{b}^{-\frac{3}{K}}-3{b}^{-\frac{5}{K}}-3{b}^{-\frac{1}{K}})\int d\tau cos[\sqrt{4\pi }({\theta }_{s}(\tau ))].\end{array}$$

The *U*^3^ term is given by

37 $$\begin{array}{c}\frac{{U}^{3}}{6}\int d\tau d{\tau }^{{\rm{^{\prime} }}}d{\tau }^{{\rm{^{\prime} }}{\rm{^{\prime} }}}[\langle cos[4\sqrt{\pi }\varphi (\tau )]cos[4\sqrt{\pi }\varphi ({\tau }^{{\rm{^{\prime} }}})]cos[4\sqrt{\pi }\varphi ({\tau }^{{\rm{^{\prime} }}{\rm{^{\prime} }}})]\rangle \\ -3\langle cos[4\sqrt{\pi }\varphi (\tau )]cos[4\sqrt{\pi }\varphi ({\tau }^{{\rm{^{\prime} }}})]\rangle \langle cos[4\sqrt{\pi }\varphi ({\tau }^{{\rm{^{\prime} }}{\rm{^{\prime} }}})]\rangle \\ \,\,\,\,\,\,\,+\,2\langle cos[4\sqrt{\pi }\varphi (\tau )]\rangle \langle cos[4\sqrt{\pi }\varphi ({\tau }^{{\rm{^{\prime} }}})]\rangle \langle cos[\varphi \sqrt{\pi }\varphi ({\tau }^{{\rm{^{\prime} }}{\rm{^{\prime} }}})]\rangle ]\\ \,\,\,\,\,\,\,\,\,\,\,\,\,=\,(\frac{{U}^{3}}{24})(6{b}^{-\frac{12}{K}}-3{b}^{-\frac{20}{K}}-3{b}^{-\frac{4}{K}})\int d\tau cos[4\sqrt{\pi }({\varphi }_{s}(\tau ))].\end{array}$$

Now we calculate $\frac{a{U}^{2}}{6}$:

38 $$\begin{array}{c}\frac{a{U}^{2}}{6}\int d\tau d{\tau }^{{\rm{^{\prime} }}}d{\tau }^{{\rm{^{\prime} }}{\rm{^{\prime} }}}[3\langle cos[\sqrt{4\pi }\theta (\tau )]cos[\sqrt{16\pi }\varphi ({\tau }^{{\rm{^{\prime} }}})]cos[\sqrt{16\pi }\varphi ({\tau }^{{\rm{^{\prime} }}{\rm{^{\prime} }}})]\rangle \\ \,-\,6\langle cos[\sqrt{4\pi }\theta (\tau )]cos[\sqrt{16\pi }\varphi ({\tau }^{{\rm{^{\prime} }}})]\rangle \langle cos[\sqrt{16\pi }\varphi ({\tau }^{{\rm{^{\prime} }}{\rm{^{\prime} }}})]\rangle \\ \,-\,3\langle cos[\sqrt{4\pi }\theta (\tau )]\rangle \langle cos[\sqrt{16\pi }\varphi ({\tau }^{{\rm{^{\prime} }}})]cos[\sqrt{16\pi }\varphi ({\tau }^{{\rm{^{\prime} }}{\rm{^{\prime} }}})]\rangle \\ \,+\,6\langle cos[\sqrt{4\pi }\theta (\tau )]\rangle \langle cos[\sqrt{16\pi }\varphi ({\tau }^{{\rm{^{\prime} }}})]\rangle \langle cos[\sqrt{16\pi }\varphi ({\tau }^{{\rm{^{\prime} }}{\rm{^{\prime} }}})]\rangle ]\\ \,=\,\frac{a{U}^{2}}{12}(3{b}^{-\frac{1}{K}}-3{b}^{-(8K+\frac{1}{K})})\int d\tau cos[2\sqrt{\pi }{\theta }_{s}(\tau )].\end{array}$$

Now we calculate *a*^2^*U* term,

39 $$\begin{array}{c}\frac{{a}^{2}\Delta }{6}\int d\tau d{\tau }^{{\rm{^{\prime} }}}d{\tau }^{{\rm{^{\prime} }}{\rm{^{\prime} }}}[3\langle cos[\sqrt{4\pi }\theta (\tau )]cos[\sqrt{4\pi }\theta ({\tau }^{{\rm{^{\prime} }}})]cos[\sqrt{16\pi }\varphi ({\tau }^{{\rm{^{\prime} }}{\rm{^{\prime} }}})]\rangle \\ -\,6\langle cos[\sqrt{4\pi }\theta (\tau )]cos[\sqrt{16\pi }\varphi ({\tau }^{{\rm{^{\prime} }}})]\rangle \langle cos[\sqrt{4\pi }\theta ({\tau }^{{\rm{^{\prime} }}{\rm{^{\prime} }}})]\rangle \\ -\,3\langle cos[\sqrt{4\pi }\theta (\tau )]cos[\sqrt{4\pi }\theta ({\tau }^{{\rm{^{\prime} }}})]\rangle \langle cos[\sqrt{16\pi }\varphi ({\tau }^{{\rm{^{\prime} }}{\rm{^{\prime} }}})]\rangle \\ \,+\,6\langle cos[\sqrt{4\pi }\theta (\tau )]\rangle \langle cos[\sqrt{4\pi }\theta ({\tau }^{{\rm{^{\prime} }}})]\rangle \langle cos[\sqrt{16\pi }\varphi ({\tau }^{{\rm{^{\prime} }}{\rm{^{\prime} }}})]\rangle ]=0.\end{array}$$

Similarly, following the same procedure used to derive the second order RG, we obtain the following equations.

$$da=\left(2-\frac{1}{K}\right)adl+2Ka{U}^{2}dl$$

Finally we write this equation in term of *Δ*,Comparing *U* terms,

$$dU=\left(2-4K\right)Udl$$

We obtain, by comparing the *ϕ* terms,

$$dK=-8{K}^{2}{U}^{2}dl$$

$$\frac{dK}{dl}=-8{K}^{2}{U}^{2}$$

We obtain, by comparing the *θ* terms,

$$\begin{array}{rcl}\frac{dK}{dl} & = & \frac{{a}^{2}}{2}\\  & = & \frac{{\Delta }^{2}}{8}\end{array}$$

Thus the full form of the equation in terms of *Δ* is

$$\frac{dK}{dl}=\frac{{\Delta }^{2}}{8}-8{K}^{2}{U}^{2}$$

Thus the complete RG equation upto third order is given by

### Modified RG equations in presence of *μ*

Now we derive the RG equations for finite chemical potential (*μ* ≠ 0). The presence of finite chemical potential yields five extra terms in the second order cumulant expansion (Eq.  (29)), of which three are vanishing. The non-vanishing contributions are from the two correlation functions, (a). $\int drd{r}^{{}^{^{\prime} }}ai\frac{\mu }{\sqrt{\pi }}$, and (b). $\int drd{r}^{{}^{^{\prime} }}i\frac{\mu }{\sqrt{\pi }}a$,

Now we calculate $ai\frac{\mu }{\sqrt{\pi }}$ term:

$$\begin{array}{c}-\frac{1}{2}\int d\tau d{\tau }^{{\rm{^{\prime} }}}(-\frac{ai}{\mu }\sqrt{\pi }\langle cos(\sqrt{4\pi }\theta (\tau )){{\rm{\partial }}}_{{\tau }^{{\rm{^{\prime} }}}}\theta ({\tau }^{{\rm{^{\prime} }}})\rangle -\langle cos(\sqrt{4\pi }\theta (\tau ))\rangle \langle {{\rm{\partial }}}_{{\tau }^{{\rm{^{\prime} }}}}\theta ({\tau }^{{\rm{^{\prime} }}})\rangle )\\ =\,\frac{ai\mu }{2\sqrt{\pi }}\int d\tau d{\tau }^{{\rm{^{\prime} }}}[\langle cos[\sqrt{4\pi }\theta (\tau )]({{\rm{\partial }}}_{{\tau }^{{\rm{^{\prime} }}}}{\theta }_{s}({\tau }^{{\rm{^{\prime} }}})+{{\rm{\partial }}}_{{\tau }^{{\rm{^{\prime} }}}}{\theta }_{f}({\tau }^{{\rm{^{\prime} }}}))\rangle \\ \hspace{9.99756pt}-\langle cos[\sqrt{4\pi }\theta (\tau )]\rangle \langle {{\rm{\partial }}}_{{\tau }^{{\rm{^{\prime} }}}}{\theta }_{s}({\tau }^{{\rm{^{\prime} }}})+{{\rm{\partial }}}_{{\tau }^{{\rm{^{\prime} }}}}{\theta }_{f}({\tau }^{{\rm{^{\prime} }}})\rangle ]\\ =\,\frac{ai\mu }{2\sqrt{\pi }}\int d\tau d{\tau }^{{\rm{^{\prime} }}}[\langle cos[\sqrt{4\pi }\theta (\tau )]{{\rm{\partial }}}_{{\tau }^{{\rm{^{\prime} }}}}{\theta }_{f}({\tau }^{{\rm{^{\prime} }}})\rangle ]\\ =-\frac{a\mu }{\sqrt{\pi }2\pi }(1-{b}^{-\frac{1}{K}})\int d\tau cos[\sqrt{4\pi }{\theta }_{s}(\tau )]\end{array}$$

Similarly one can calculate the, $\int drd{r}^{^{\prime} }\frac{i\mu }{\sqrt{\pi }}a$.

Finally, combination of these two terms (*a* + *b*) yields

$-\frac{a\mu }{\pi \sqrt{\pi }}(1-{b}^{-\frac{1}{K}})\int d\tau cos\left[\sqrt{4\pi }{\theta }_{s}(\tau )\right.$, as a consequence of it *a* term modify to

$$\bar{a}=a({b}^{2-\frac{1}{K}})-\frac{a\mu }{\pi \sqrt{\pi }}({b}^{2}-{b}^{2-\frac{1}{K}}).$$

Finally we obtain the modified 2nd order and 3rd RG equations in presence of *μ* from the rescaled *a* term as

$$\begin{array}{rcl}\bar{a} & = & a({b}^{2-\frac{1}{K}})-\frac{a\mu }{\pi \sqrt{\pi }}({b}^{2}-{b}^{2-\frac{1}{K}})+\left(\frac{{a}^{3}}{24}\right)\left(6{b}^{2-\frac{3}{K}}-3{b}^{2-\frac{5}{K}}-3{b}^{2-\frac{1}{K}}\right)\\  &  & +\frac{a{U}^{2}}{12}\left(3{b}^{2-\frac{1}{K}}-3{b}^{2-(8K+\frac{1}{K})}\right)\end{array}$$

The correlation functions for finite *μ*, only show up for the RG equations of $\frac{da}{dl}$ because only these two correlation functions give non vanishing contributions. Thus we have the modified 2nd order and 3rd order RG equation in terms of *Δ* as4041
